# Plant melanin as a microcapsule wall component for flaxseed oil oxidative stability improvement

**DOI:** 10.1038/s41598-025-24023-6

**Published:** 2025-11-17

**Authors:** Grzegorz Dąbrowski, Iwona Konopka, Dorota Ogrodowska

**Affiliations:** Chair of Plant Food Chemistry and Processing, Faculty of Food Sciences, University of Warmia and Mazury in Olsztyn, Pl. Cieszyński 1, Olsztyn, 10-726 Poland

**Keywords:** Flaxseed oil, Encapsulation, Melanin, Oxidation, Oil-powders, Oils, Plant sciences

## Abstract

Flaxseed oil is very susceptible to oxidation due to high α-linolenic acid (ALA) content. Encapsulation is a widely used method to stabilize ALA. The aim of the current study was to compare the oxidative stability of flaxseed oil capsules made using black cumin and black sesame melanin extract. The prepared emulsions were spray-dried, and the particle size, shape, color, Carr’s index, and oxidative stability of the obtained powders were measured. Oxidative stability was measured using the Rancimat, DPPH, and ABTS tests and by measuring the peroxide value and thiobarbituric acid reactive substances (TBARS) after UV irradiation of the powders. The results showed that the addition of melanin does not determine morphology and slightly enhanced the flowability of the powders. The longest induction period was recorded for 1% of black sesame melanin in the wall material. Black sesame melanin was also a good protective agent of flaxseed oil against oxidation under the influence of UV irradiation (the peroxide value was about 30% lower in the variant with 1% addition compared to the control sample) and showed a better antioxidant capacity in the ABTS assay. On the other hand, black cumin melanin showed a higher antioxidant capacity in the DPPH test. The results confirm the possibility of adding plant melanin to the material encapsulating flaxseed oil where they can act as light shielding and antioxidant agent.

## Introduction

The term „melanin” is used to describe a group of pigments with a specific set of properties. Its extraordinary properties include one of the highest refractive index values known in nature and broad absorption spectrum^1^. Melanin is commonly found in all life forms in nature, where it plays such roles as pigmentation, radical scavenging, radiation protection, and thermal regulation. In addition, there is still a lack of knowledge about the biosynthesis of this biopolymer. There are a lot of variables affecting this process and melanin structure, as different starting monomers and broad interactions holding their molecules together, as covalent bonding, hydrogen-bonding, cation − π, and aromatic interactions. The complex nature of this compound (polymers with molecules ranging in size from nanometers to microns) and insolubility in most used solvents have made its properties difficult to study^2^.

**Fig. 1:**
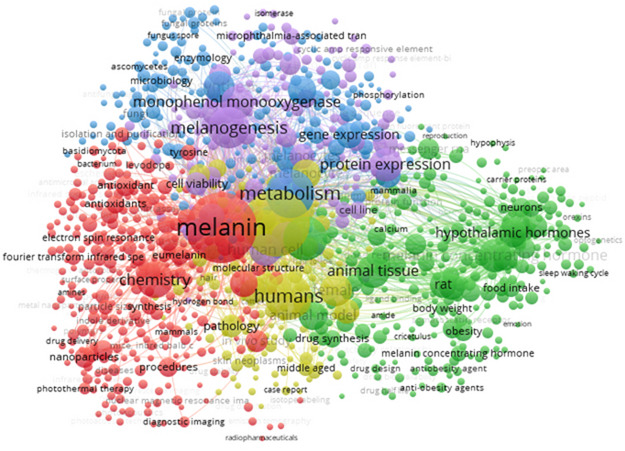
Viewability of term “melanin” in scientific sources since 2000 year. The size of the circle represents the frequency of co-occurrence of a given keyword. Image generated based on the Scopus database with the use of VOSviewer 1.6.20 software.

Analyzing literature data with the use of Scopus database, it was found, that prompt “(TITLE (melanin) AND NOT TITLE (review))” have returned 4945 documents published from year 2000 until present. As it is presented in Fig. 1., there are a lot of keywords related with chemical analysis and health co-occurring with the “melanin” word in titles and keywords of analyzed documents. Health-related keywords suggest mainly the research concentrated on anti-obesity activity of this compound while chemical analyses are focused on antioxidant activity including radical scavenging activity. For example, Oh et al.^3^ found that fungal melanin had antioxidant activity like ascorbic acid but higher than reduced glutathione. Cited research was focused on cosmetical application of melanin in sunscreen products in which, melanin was classified as promising active ingredient. Also, research conducted by Sheefaa et al.^4^ presented significant antioxidant activity of melanin extracted from marine microorganisms. Next study conducted by Bozhuyuk et al.^5^, confirmed antioxidant and sunscreen properties of melanin. Cited authors studied the properties of microbial melanin produced by *Scolecobasidium musae*. They concluded that melanin can both scavenge the stable free radical DPPH to an extent comparable to known phenolic antioxidants and protect against UV radiation, demonstrating high stability to the influence of this factor.

Most studies concentrate on the characterization of microbial melanin. There is still a lack of information in the literature on the characteristics of plant-derived melanin. Some documents show the presence of melanin in watermelon seeds^6^, sesame seeds^7^, black cumin seeds^8^ or sunflower testae^9^. There are also reports about melanin-like pigments in chestnut shells^10^, black mustard coats, rapeseed coats^11^, black garlic^12^ as well as *Ipomoea tricolor* coats^13^.

Some of these plants have a high oil content that can be pressed or extracted. Melanin’s presence in press cake makes it a very valuable by-product of the oil industry. Defatted seeds are a good substrate for melanin isolation. As mentioned earlier, melanin is poorly soluble in most known solvents. However, it solubilizes well in alkaline solutions while in low pH, it tends to precipitate. These properties are useful in melanin isolation because alkaline solutions may be used for extraction. Next, acidification may be applied for melanin precipitation and purification^7,14^.

Due to known antioxidant and anti-UV properties of melanin^15^, they seem to be a good protector for oxidatively unstable edible oils. One of the representatives of such oils may be flaxseed oil. This oil contains high amounts of α-linolenic acid (ALA), an n-3 fatty acid recommended in the diet. In the human body, this fatty acid is a precursor in the biosynthesis of essential, long-chain, unsaturated fatty acids, such as eicosapentaenoic acid (EPA) and docosahexaenoic acid (DHA). Flaxseed oil may be an excellent dietary choice for people who avoid fish and seafood products^16^. Despite the nutritional value, content of PUFAs exceeding 70% in fresh flaxseed oils^17^ make them very susceptible to oxidation process and limits their storage stability.

Due to this fact, there were successful attempts of flaxseed oil encapsulation with the use of different wall materials. Up to date were tested maltodextrin (MD) with whey protein concentrate (WPC), MD with gum Arabic (GA), octenyl succinic anhydride (OSA) starch and MD, OSA starch and nutriose, OSA starch with inulin^18,19^; MD with GA, WPC, and modified starch (Hi-Cap 100TM and Capsul TA^20^; flax mucilage^21^; MD with WPC and sodium alginate (SA) [22]; MD with sodium caseinate (SC) and transglutaminase^23^, and various vegetable proteins, such as chickpea, lentil, flaxseed, soy, pea, etc^24^. Several compositions abundant in antioxidants significantly reduced the formation of primary and secondary oil oxidation products. Many natural antioxidants are found in food, including water-soluble bioactive substances such as citrates, norbixin, betalains, ascorbic acid, phenolic compounds such as flavanoids and anthocyanins, and lipid-soluble components such as carotenoids, tocopherols, and terpenoids^25^. Many of these have been used as microcapsule wall materials, either in their pure form or as preparations/extracts. Maltodextrin and gum Arabic also protect powders from oxidation during storage at low humidity^26^.

The only challenge in application of melanin in oils stabilization may be their insolubility in hydrophobic environments. A good solution for stability issues with flaxseed oil and melanin solubility may be the use of flaxseed oil microcapsules in which melanin will be component of the wall. In such conditions, there is no solubility in oil needed, while there is a need to dissolve them for a short while in water, which will be a component of the emulsion before the actual microencapsulation process. Plant melanin is insoluble in pure water, while alkaline solutions are not good for food applications, especially in the presence of oils, where they could cause saponification reactions. The solution to this problem may be the neutralization of alkaline melanin solution just before emulsification. Such an approach was applied for example in a study assessing anti-ulcerogenic effects of melanin extracted from *Nigella sativa* L. Mentioned solutions were added to the diets of experimental animals^27^.

This study aimed to develop oxidatively stable flaxseed oil capsules by the use melanin extracted from black cumin and black sesame seeds. To the best of the authors’ knowledge, this is the first work to use melanin to microencapsulate edible oil.

## Materials and methods

### Materials

Black sesame and black cumin seeds were purchased from the local market. Flax seeds were cultivated at the Production and Experimental Plant “Bałcyny” LLC in Bałcyny (Poland, Warmian-Masurian Voivodeship, 53.35°N, 19.51°E). These seeds were used for cold pressing of flaxseed oil with the use of an IBG Monforts & Reiners, Komet CA59G (Mönchengladbach, Germany) laboratory screw press equipped with a 4 mm diameter nozzle. The pressing process was conducted at a temperature below 45 °C. The obtained oil samples were purified via centrifugation at 12,333×g using an Eppendorf 5810R centrifuge (Eppendorf, Hamburg, Germany). Obtained oil was stored at −20 °C until emulsion preparation. The fatty acid composition of the obtained flaxseed oil was as follows: α-linolenic (58.8%), oleic (17.0%), linoleic (15.9%), palmitic (4.6%), and stearic (3.3%). The peroxide value of obtained oil was 0.8 meq O_2_/kg. Maltodextrin was from Pepees S.A. Company (Łomża, Poland), and gum Arabic from Kremer Pigmente GmbH&Co.KG (Aichstetten, Germany).

### Melanin isolation

Black sesame and black cumin seeds were defatted in 2-step process. The first was cold pressing, using the same equipment and under the same conditions as in the previous point. Next, the press cake was milled and extracted in a Soxhlet apparatus according to ISO 659:2010 standard^28^. Defatted seeds were next subjected to melanin isolation. Briefly, 5 g of defatted seeds were weighed into falcon tube and 40 mL of 1 M NaOH was added. Extraction was performed by rotation of tubes at room temperature by 24 h, at 30 rpm in Multi RS-60 Rotator (Biosan, Riga, Latvia) and next centrifugation at relative centrifugal force 12,333×g by 10 min using an Eppendorf 5810R centrifuge (Eppendorf, Hamburg, Germany). Extract was decanted through a quantitative soft paper filter. The precipitate obtained was extracted twice for 30 min with new portions of 1 M NaOH. Combined extracts were acidified to pH = 2 with the use of 6 M HCl and precipitated by 24 h. Next, samples were centrifuged and purified by re-dissolving in 1 M NaOH and precipitated with the same conditions as described earlier. The resulting precipitate was washed with deionized water until neutral pH was obtained in the presence of indicator paper. Next, wet melanin was dried in Memmert vacuum dryer at 50 °C and 50 mbar to a constant mass. The dry melanin was then ground into powder in a laboratory mortar and stored in −20 °C until the emulsion was prepared.

### UV-VIS spectrum analysis

To determine the UV–VIS spectra of the isolated melanins, 30 mg of melanin powders were dissolved in 2 mL of 1 M NaOH and diluted to 50 mL with deionized water. 50 µL of the resulting solutions were added into wells of 96-well UV microplate together with 250 µL of deionized water. Resulting solutions were scanned with the use of a FLUOstar Omega apparatus (BMG Labtech GmbH, Ortenberg, Germany) in wavelength range from 200 to 1000 nm.

### Emulsions Preparation

Melanin solutions were prepared according to the study presented by El-Obeid et al.^25^. Water for each variant of the emulsion was alkalized to pH = 12.5 using 1 M NaOH. Then a weighed amount of melanin powder (0.5 or 1% of the total weight of wall materials) was added, and the mixture was shaken until the melanin was completely dissolved. Immediately before adding to the emulsion, the solution was neutralized with 1 M HCl. Firstly, the samples were prepared by mixing wall materials (150 g of maltodextrin and 50 g of gum Arabic) and water with melanin (700 ml of neutralized melanin solution) using a Thermomix (Vorwerk, Germany) at 9000 rpm for 10 min. Next, lipid fraction (100 g of flaxseed oil) was added, and mixed for another 5 min. Obtained emulsions were then homogenized (first at 24 MPa and then at 4 MPa) using a Panda 2 K laboratory homogenizer (GEA Niro Soavi, Parma, Italy). The composition of the wall was optimized in our previous study^18^.

### Encapsulation process and powder characteristics

Microcapsules were prepared from whole emulsion prepared according to previous point with the use of a pilot-plant spray-dryer (A/S Niro Atomizer, Copenhagen, Denmark). The drying parameters were controlled to maintain an inlet temperature of 130 °C and an outlet temperature of 90 °C, and the feed flow rate was 77 mL/min. Particle size distribution of each powder was determined with the use of a Mastersizer 2000 (Malvern Instruments Ltd., Worcestershire, UK). It was characterized by Sauter mean diameter D[3,2] (the surface weighted mean diameter), De Brouckere mean diameter D[4,3] (the volume weighted mean diameter), and specific surface area (SSA). Additionally, the width of the distribution (*Span*) was calculated based on the distribution of diameters of the droplets at which 90%, 50% or 10% of the sample was smaller than the size measured (Dx (10), Dx (50), and Dx (90), respectively) according to our previous study^18^. Carr’s index was calculated based on bulk and tapped density measurement according to our previous study^19^.

### Encapsulation efficiency and yield analysis

Firstly, surface oil was extracted from 2 g of powder with the use of 15 mL of n-hexane for 1 min at room temperature. Then, the solvent was filtered, and evaporated using a rotary evaporator (Büchi Labortechnik AG, Flawil, Switzerland). Obtained oil was weighed, and the surface oil content was expressed as a percentage of total oil used to emulsion preparation. The encapsulation efficiency was calculated according to our previous study^18^. Encapsulation yield was calculated by determining the mass ratio of the obtained powder to the mass of dry ingredients introduced into the emulsion and expressed as percentage.

### Color analysis

Melanin powders and microcapsule powders were placed in flat, round cylinders, while the seeds were poured onto a flat white surface in such a way that they did not touch each other. Image was recorded with the use of a high resolution, low-noise CCD Nikon DXM 1200 color camera (Nikon Inc., Melville, USA), saved as “.lim” files and analyzed by LUCIA G v. 4.8 software (Laboratory Imaging, Prague, Czech Republic). The frame grabber was at a resolution of 1,280 × 1,024 pixels. The light source was provided by 4 BOB OM optical lamps with 4,100 W (60 kLx) halogen bulbs (OSRAM firm, Poland), with a color temperature of 3,000 K (warm white). The lamps were attached at a 45° angle from above the object on a par with the camera. Before the measurements, the testing set was calibrated in accordance with the international whiteness standard using a calibration plate. Color was expressed in CIE L*a*b* model (lightness, greenness-redness, blueness-yellowness) in which the L* component was in the range 0–100%, a* and b* components were in the range from − 120 to + 120.

### Oxidative stability analysis

The oxidative stability of flaxseed oil and powders was measured with the use of Rancimat apparatus 743 (Metrohm, Herisau, Switzerland), according to our previous study^18^. Briefly, 2.5 g of powder was weighed into a reaction tube and placed in a thermostated heating block. The temperature was set at 110 °C and air flow rate was 20 mL/min. Volatile oxidation products were measured conductometric, and the time that elapsed until these oxidation products appeared was saved as the induction period describing the stability of powders.

### Powder stability under UV irradiation

The irradiation of powders was performed based on Belhoussaine et al.^29^ with some modifications. Briefly, an amount of 20 g of each powder was weighed into a glass Petri dish creating a thin-layer not exceeding 1 mm of thickness. Samples were put in a light-proof chamber and placed 70 cm under a stand lamp equipped in 2 Philips 36 W/G36 T8 UV fluorescent lamps with wavelength of 254 nm. Irradiation was carried out for 8 h. After the irradiation, powders were collected to a glass beaker and subjected for total lipids extraction according to Folch procedure^30^. Next, the peroxide value of extracted oils was measured according to ISO 3960:2012 standard^31^. Powders that were not irradiated were also analyzed for peroxide value in the same manner.

Powders were also subjected to TBARS analysis, according to Lee and Yoon^32^ with some modifications. Briefly, to 2 g of powder, 8 mL of deionized water was added, and sample was homogenized with the use of VCX750 ultrasound device (Sonics & Materials Inc., Newtown, CT, USA). Next, 6 mL of TBA reagent and 3 mL of 20% trichloroacetic were added. Samples were then mixed and heated in boiling water bath for 20 min. Next, samples were filled up to 20 mL of total volume, filtered through 0.20 μm syringe filters and subjected to absorbance measurement at a wavelength of 533 nm with the use of FLUOstar Omega apparatus. TBARS was expressed as µM MDA/g of powder based on external calibration curve.

### Antioxidant potential

The antioxidant capacity of melanin was measured spectrophotometrically using DPPH radical (2,2-diphenyl-1-picrylhydrazil) and ABTS (2,2’-azino-bis(3-ethylbenzothiazoline-6-sulfonic acid diammonium salt).

In DPPH method, 50 µL of solutions prepared same as in UV-VIS analysis was taken into wells of 96-well test microplate with 250 µL of a 0.36 mM methanol solution of DPPH. Absorbance was measured after 16 min of the reaction at 515 nm using the FLUOstar Omega microplate reader. A blank sample was prepared in the same manner by replacing the melanin extract with the same volume deionized water with corresponding amount of NaOH as in melanin solutions. There was also prepared vitamin C solution in the same concentration as melanin solution to compare the antioxidant activity of melanin with.

In ABTS method, 10 µL of solutions prepared same as in UV-VIS analysis was taken into wells of 96-well test microplate with 290 µL of a 7 mM methanol solution of ABTS. Absorbance was measured after 5 min of the reaction at 734 nm using the FLUOstar Omega microplate reader. Blank sample and vitamin C solution were prepared in the same way and measured according to previously described conditions.

Antioxidant capacity in both methods was expressed as µmol Trolox (TE) per 1 g of melanin/vitamin C.

### Statistical analysis

All the analyses were performed in at least triplicate, and the values are reported in the tables as mean ± standard deviation. Statistical analysis of the results was performed using Statistica 13.3 software (TIBCO, Palo Alto, CA, USA), which was employed for analysis of variance (ANOVA) with Tukey’s test for homogeneous groups. All the calculations were conducted at a significance level of *p* ≤ 0.05.

## Results and discussion

### Shape and particle size of powders and preparations of melanin

**Fig. 2:**
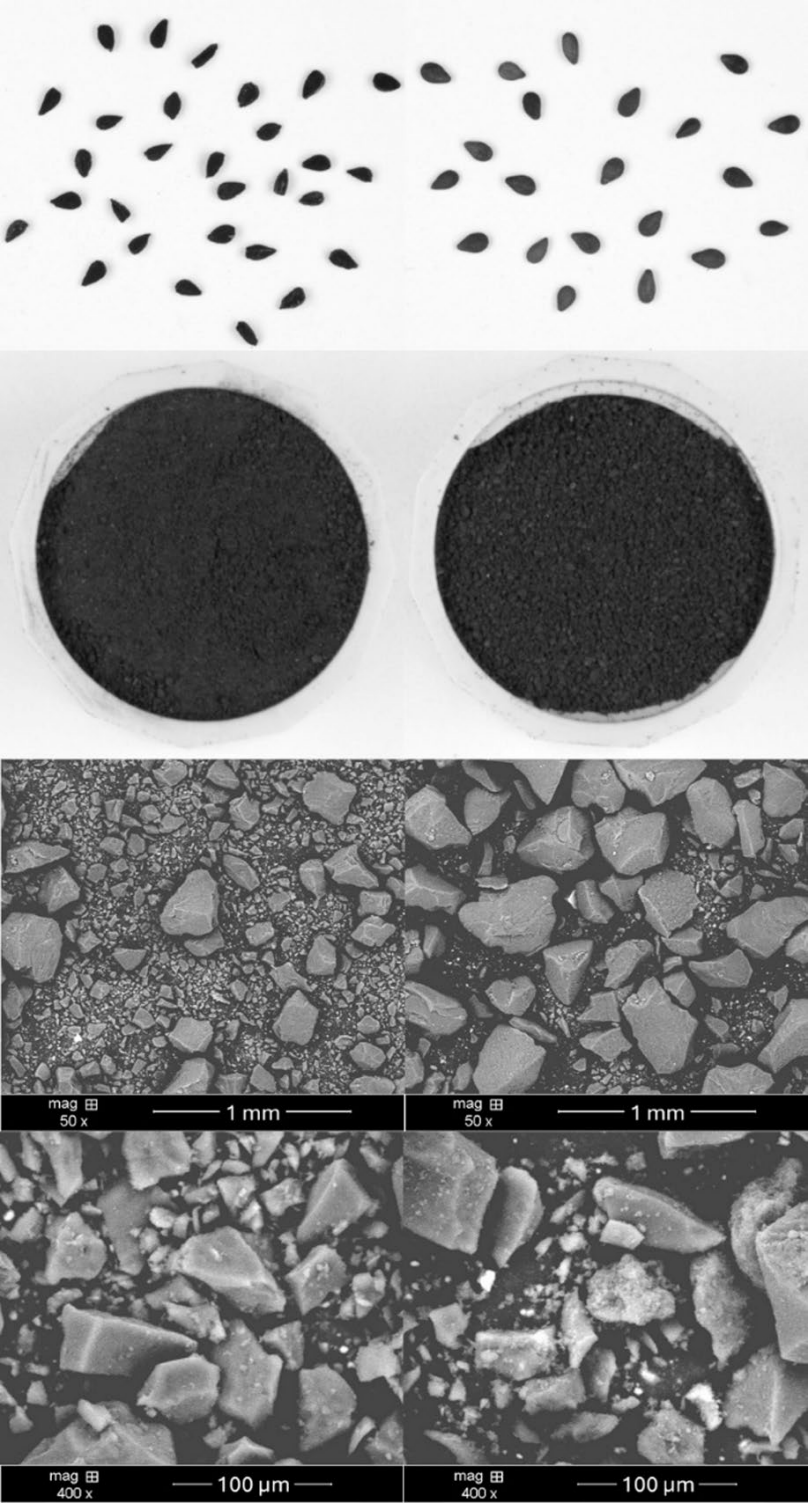
Macroscopic and SEM pictures of black cumin (left side) and black sesame (right side) isolated melanin.

Macroscopic and SEM images of melanin preparations are presented in Fig. 2.

SEM images are typical of powders, showing their flake-like shape. The size and shape of both are similar and are mainly due to the method of crushing them. In both cases, the same grinding method was used, but in the case of black cumin seeds a significant fraction of small particles was obtained.

**Fig. 3:**
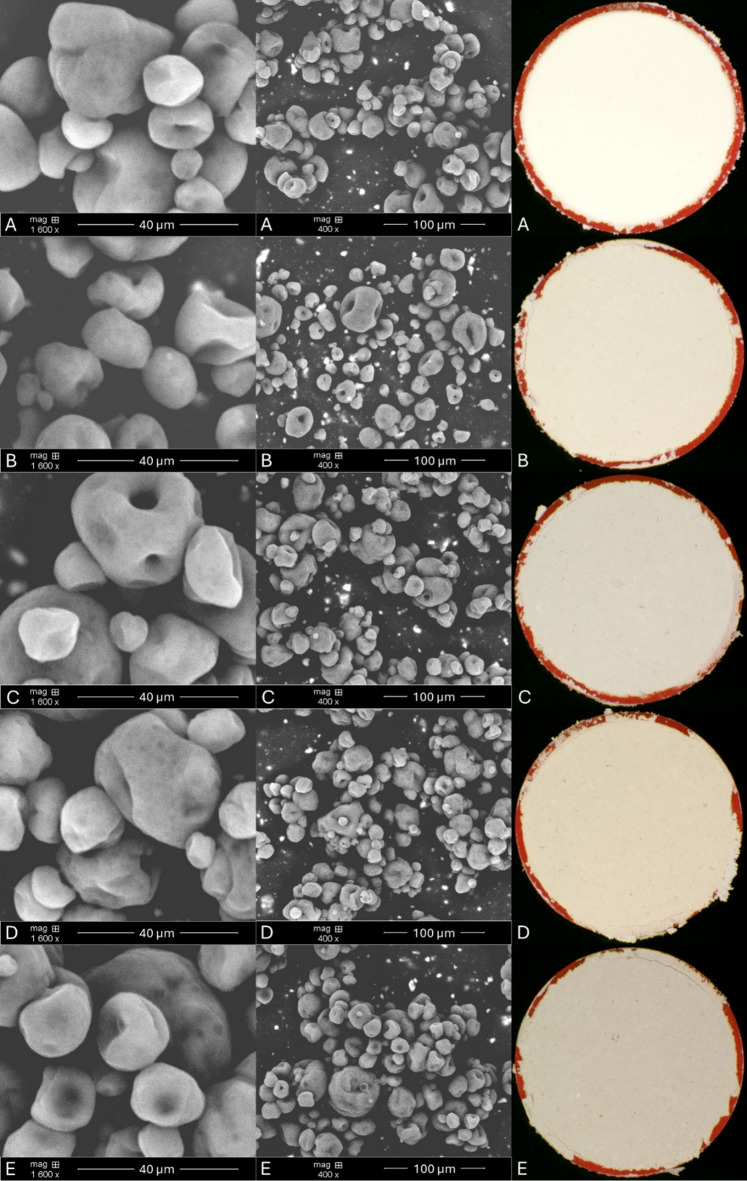
SEM and macroscopic images of obtained powders (A – control powder, B – black cumin 0.5%, C – black sesame 0.5%, D – black cumin 1%, E – black sesame 1%).

SEM images of obtained powders (Fig. 3) showed morphology of microcapsules, similar in size and shape in all samples as was observed in the particle size results (Table 1).

**Table 1:** Powders particle size (µm) and Carr index (%).

	Dx (10)	Dx (50)	Dx (90)	D [4,3]	D [3,2]	SSA	Span	Carr index
Control powder	15.8 ± 0.1 c	31.9 ± 0.2 bc	73.5 ± 0.3 ab	47.7 ± 1.4 a	28.3 ± 0.2 c	202 ± 1 a	1.81 ± 0.01 a	35.0 ± 0.2 c
Black cumin 0.5%	14.5 ± 0.1 a	29.5 ± 0.3 a	68.6 ± 2.1 a	43.2 ± 5.2 a	26.1 ± 0.3 a	219 ± 3 c	1.83 ± 0.05 a	32.8 ± 0.7 ab
Black sesame 0.5%	15.2 ± 0.1 b	31.2 ± 0.2 b	75.1 ± 1.9 b	49.8 ± 7.3 a	27.6 ± 0.2 b	207 ± 2 b	1.92 ± 0.05 a	33.8 ± 0.7 bc
Black cumin 1%	15.3 ± 0.1 b	32.4 ± 0.4 c	92.2 ± 3.3 d	56.4 ± 9.3 a	28.4 ± 0.3 c	201 ± 2 a	2.38 ± 0.07 c	33.2 ± 0.2 abc
Black sesame 1%	15.3 ± 0.1 b	31.7 ± 0.2 bc	82.4 ± 1.1 c	46.8 ± 1.0 a	28.0 ± 0.1 bc	204 ± 1 ab	2.12 ± 0.04 b	31.6 ± 0.3 a

a, b, … – Means in the same column for all variants followed by different letters are significantly different (*p* ≤ 0.05); Values are mean ± SD (*n* = 3).

Their structure is spherical with pores and cavities. They are also observed with little holes inside but despite this the structure of microcapsules is still proper for protecting the core material. The images show that the addition of melanin does not determine the morphology of the powders. It depends rather on the coating material used and the temperature of the spray drying process. In our study, an inlet temperature of 130 °C was used which considering the cooling effect of water evaporation, it does not contribute to the oxidation of unstable flaxseed oil. Shamaei et al.^33^ reported that the walnut oil capsules obtained in higher temperature (e.g. capsules obtained at 180 °C) had smoother surface in comparison to those obtained at lower (140 °C) under the same nozzle pressure. On the other hand, in a study conducted by Francisco et al.^34^ morphology of microcapsules obtained in 180 °C did not present visual differences from the one produced at 150 °C and the surface depression are related to fast water loss during drying.

The mean diameters of particles (median Dx (50)) of powders ranged from 29.5 (black cumin 0.5%) to 32.4 μm (black cumin 1%). The diameter Dx (10) was in the range from 14.5 (black cumin 0.5%) to 15.8 μm (control powder) and diameter Dx (90) from 68.6 (black cumin 0.5%) to 92.2 μm (black cumin 1%). Domian et al.^35^ presented similar results Dx (50) = 33.3 and 35.9 μm for the microcapsules of flaxseed oil coated by composition of wheat dextrin soluble fiber with soy protein isolate and pea protein isolate, respectively. The volume-weighted mean particle diameter D [4,3] was smaller in sample with 0.5% black cumin addition (43.2 μm) when to compare to sample with 1% black cumin addition (56.4 μm), although it was no statistical differences between them.

**Fig. 4:**
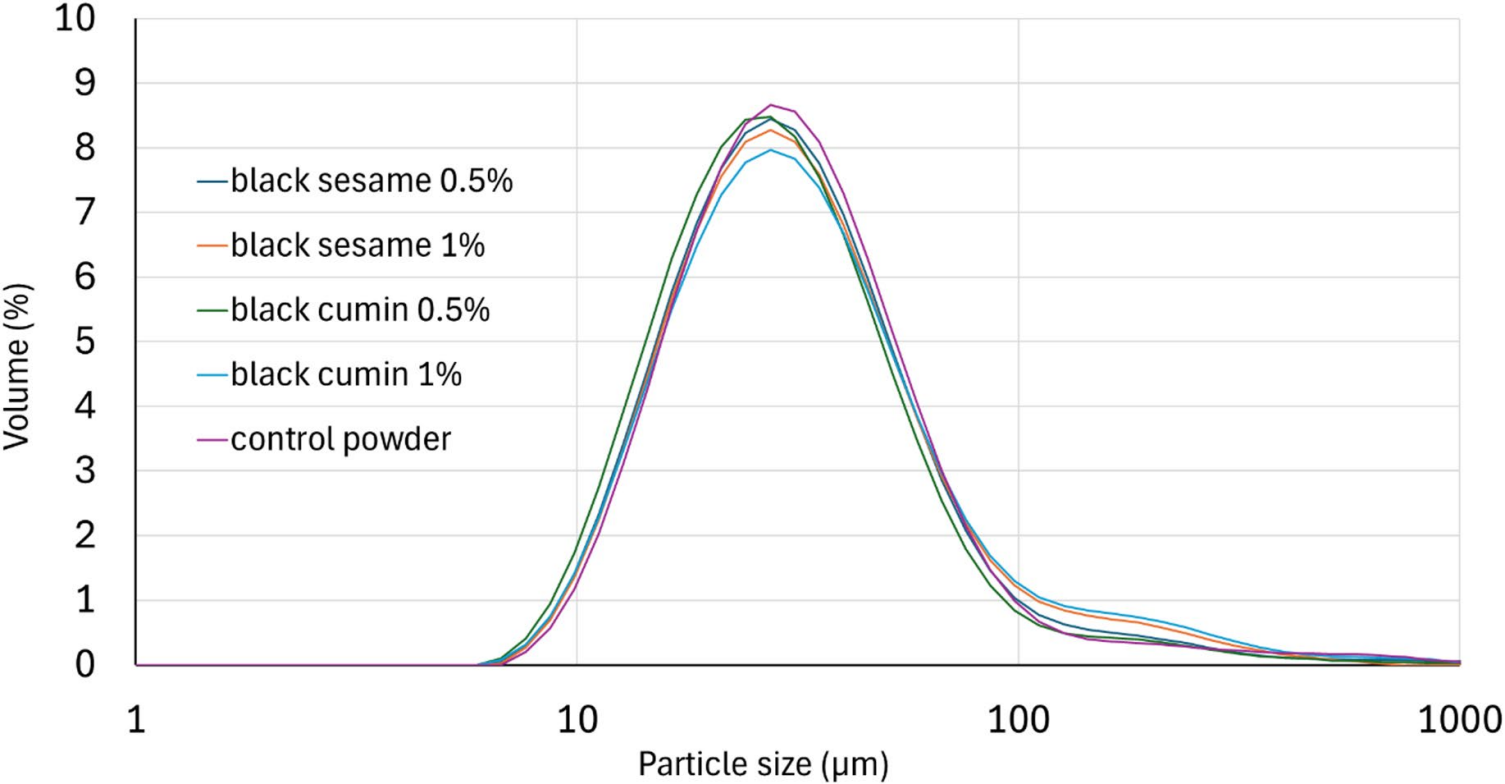
Particle size distribution (by volume) of obtained powders.

The microcapsules presented a monomodal size distribution (Fig. 4). This type of distribution indicates that the samples had a homogenous particle size. Tatar et al.^36^ using maltodextrin and gum Arabic as a coating for fish oil also obtained a monomodal particle distribution.

### The carr’s index

The Carr’s index points on the flow behaviour of the powders. In general, a higher Carr’s index shows enhanced powder compressibility and decreased flowability^37^. Results for the prepared powders are presented in Table 1. Control sample has got value of 35.0, while all powders with melanin preparations reached lower values within the range of 31.6–33.8. It points that melanin addition slightly increased powder flowability and decreased its compressibility. Obtained results show on very cohesive flow of prepared powders^38^. Even higher cohesiveness of powders made of maltodextrin and gum Arabic was found by Mehrali at al^39^..

### Encapsulation efficiency (EE) and yield (EY)

Encapsulation efficiency is presented in Table 2. It varies from 82.7% for control powder to 87.4% for powder with 0.5% of black cumin melanin. There is no straight dependence between amount or type of melanin added and encapsulation efficiency. Generally, powders with 0.5% addition of melanin had higher encapsulation efficiency.

**Table 2:** Encapsulation efficiency (EE) and yield (EY) of obtained powders.

	EE (%)	EY (%)
Control powder	82.73 ± 0.08 a	82.70 ± 0.12 a
Black cumin 0.5%	87.45 ± 0.13 d	86.66 ± 0.49 c
Black sesame 0.5%	87.25 ± 0.24 d	86.58 ± 0.67 c
Black cumin 1.0%	83.67 ± 0.22 b	82.98 ± 0.11 a
Black sesame 1.0%	85.72 ± 0.04 c	84.84 ± 0.20 b

a, b, … – Means in the same column for all variants followed by different letters are significantly different (*p* ≤ 0.05); Values are mean ± SD (*n* = 3).

In the literature on microencapsulation of oils, there is a wide range of values for this parameter. For example, Karaaslan et al.^40^ also used maltodextrin and gum Arabic as wall materials to encapsulate oil from pepper seeds. Depending on proportions of wall components, they presented encapsulation efficiency varying from 67.45 to 87.14%. On the other hand, Karrar et al.^41^ obtained encapsulation efficiency of gurum seed oil with the use of maltodextrin and gum Arabic at level 97.38% which was the highest value in cited study, higher in comparison to microcapsules with wall containing whey protein isolate. In a study presented by Rezvankhah et al.^42^ wall of microcapsules composed of maltodextrin, gum Arabic and whey protein concentrate. All obtained powders indicated EE of higher than 90%. As the literature shows, many factors influence the efficiency of oil encapsulation. Our results showed that the addition of plant melanin may improve encapsulation efficiency of flaxseed oil. There are some reports indicating their ability to make complexes with proteins and carbohydrates^43^. Unfortunately, the number of sources on melanin binding to carbohydrates is limited and mainly focuses on the binding of microbial melanin to cell wall polysaccharides^44,45^. This feature undoubtedly needs more scientific focus and if confirmed, it may be a cause of better bonding of the microcapsule wall, which results in better oil encapsulation efficiency.

The encapsulation yield determined for the control powder was 82.70%. The addition of melanin slightly increased the values ​​of this encapsulation parameter (up to 86.66% for the 0.5% black cumin preparation). The higher encapsulation yield may be due to the lower surface oil content, which increased the flowability of the obtained powders and lowered losses in the dryer. Similar values ​​for the encapsulation of linseed oil by spray drying were previously presented by Dabiri Mohaved et al.^46^.

### Color of seeds, extracted melanin, and flaxseed oil powders and melanin UV-VIS spectrum

Color values of studied samples are presented in Table 3. Theoretically, the L* value can change from 100 (absolute white) to 0 (absolute black). It was found that L* values of both used seeds differed significantly (*p* < 0.05). In this regard blacker were black cumin seeds (51.40 vs. 58.20). L* values were the lowest for melanin with values practically at the same level of 47.36 and 46.49. The control powder was close to absolute white with an L* value of 99.31. The addition of 0.5 and 1.0% melanin gradually reduced the L* value to the lowest level for 1% of sesame melanin addition (89.60). All samples exhibited negative values of a*, which points on shade of green. The greenest shade was noted for extracted melanin with values − 7.79 and − 9.96 for black cumin and sesame seeds, respectively. As expected, the lowest shade of green was found for powders, and these values were in the range of −1.06 – −0.08. Although b* value of both seeds was similar (12.72 and 12.06) and statistically indistinguishable (*p* < 0.05), isolated melanin highly differed, with significantly higher yellow shade for preparation extracted from black cumin seeds (27.41 vs. 19.09). It resulted in significantly higher yellow shade of powders made with black cumin melanin. Yellowness changed also in dose-dependent manner. ΔE difference between control sample and powders with melanin reached the highest value of 11.44 for powder with 1% of sesame melanin. It was found that ΔE below 1 is not perceptible by human eyes, in range 1–2 is perceptible through close observation, in range 2–10 is perceptible immediately, and in range 11–49 points colors are more similar than opposite^47^. In general, low standard deviation values ​​indicate uniformity of color in the prepared powders. Dissolving the melanin at pH 12.5 and neutralizing it to pH 7 before adding it to the emulsion resulted in a uniform melanin solution, which is further attributed to its negative charge^48^.

**Table 3:** Color values in a CIE L*a*b* model of obtained powders, isolated melanin, and used seeds.

	L*	a*	b*	ΔE
Powders				
Control	99.31 ± 0.08 e	−1.06 ± 0.04 a	3.68 ± 0.16 a	-
Black cumin 0.5%	95.58 ± 0.07 d	−0.44 ± 0.09 b	5.36 ± 0.04 c	5.75 ± 1.45 a
Black sesame 0.5%	92.86 ± 0.09 b	−0.19 ± 0.09 c	4.33 ± 0.04 b	7.82 ± 0.09 b
Black cumin 1.0%	93.19 ± 0.00 c	−0.30 ± 0.00 bc	7.10 ± 0.00 e	9.40 ± 0.00 b
Black sesame 1.0%	89.60 ± 0.13 a	−0.08 ± 0.14 c	5.96 ± 0.15 d	11.44 ± 0.18 c
Melanin				
Black cumin	47.36 ± 0.77 a	−7.79 ± 0.12 b	27.41 ± 1.07 b	-
Black sesame	46.49 ± 0.47 a	−9.96 ± 0.11 a	19.09 ± 0.86 a	-
Seeds				
Black cumin	51.40 ± 1.91 a	−6.95 ± 0.89 a	12.72 ± 1.62 a	-
Black sesame	58.20 ± 3.82 b	−5.58 ± 1.10 b	12.06 ± 1.78 a	-

a, b, … – Means in the same column for all variants followed by different letters are significantly different (*p* ≤ 0.05); ΔE – total color difference; Values are mean ± SD (*n* = 3).

The color of the control powder was like the color of other powders made with carbohydrates as wall components^49^. Slightly less white and more intensely colored are powders made with the use of various proteins, for example from lentil seeds^50^.

**Fig. 5:**
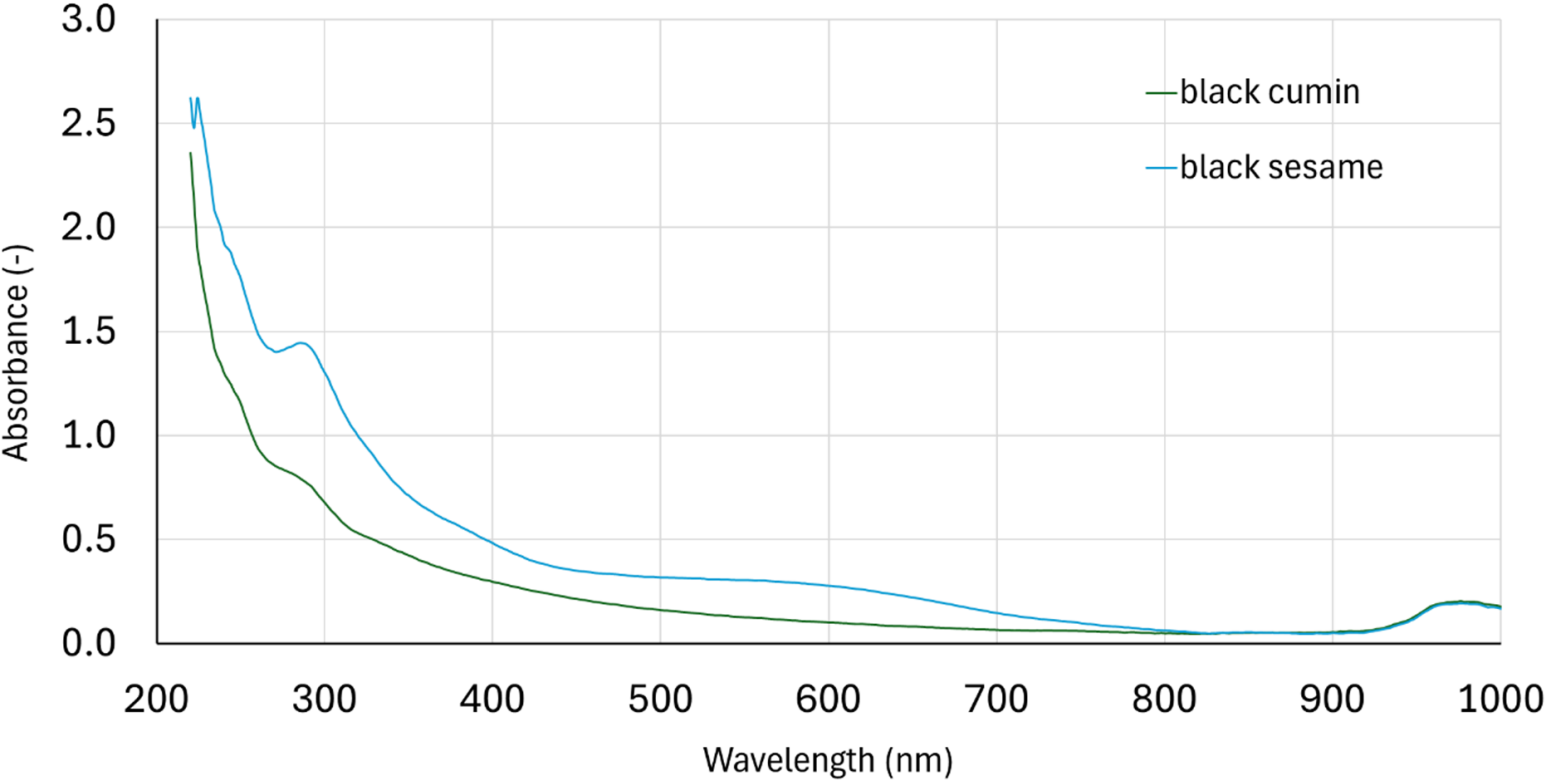
UV-VIS spectrum of extracted melanin.

In current study UV-VIS spectrum in range 200–1000 nm of extracted melanin was also determined (Fig. 5). A gradual decrease in absorption was observed in this range. The high absorption of UV radiation by melanin is most likely due to complex conjugated molecules in the melanin structure that absorb and scatter UV radiation photons^51^. It was noted also one characteristic absorption peak at a wavelength of approximately 290 nm. This peak can be related to several impurities like protein^52^ or nucleic acid or lipid^51^. A similar spectrum was previously presented for sepia melanin^51^. The destruction of the close association of proteins and other biological components with melanin can be achieved using boiling acids or bases for several hours, followed by successive washing steps of the precipitate. Unfortunately, melanins in such conditions are decomposed to a significant extent, especially through extensive decarboxylation^51^. It’s worth knowing that similar impurities can be found in other wall materials such as commercial maltodextrin and gum Arabic.

### Peroxide value and TBARS

To test the antioxidant properties of melanin preparations, the powders were exposed to UV irradiation. It was also confirmed that no significant oxidative changes occurred in the powders before irradiation (peroxide value for fresh powders did not exceed 0.8 meq O_2_/kg oil). In turn, after eight hours of exposure to UV irradiation, the flaxseed oils extracted from the powders were heavily oxidized (Table 4). It was stated that the peroxide value of the control sample was 69.1 meq O_2_/kg oil. Powders prepared with melanin as wall components at 0.5% concentration exhibited PV with values of 70.0 and 59.2 meq O_2_/kg oil for black cumin and black sesame seeds, respectively. It was emphasized that melanin from black sesame in this concentration has antioxidant properties, while black cumin does not. Higher antioxidant activity was noted for melanin added at 1% concentration. At this concentration both black cumin and black sesame melanin significantly protected flaxseed oil oxidation. The degree of oil oxidation in these cases was 55.2 and 47.7 meq O_2_/kg fat, respectively. This indicates that black sesame seed melanin is a better antioxidant than black cumin melanin and that the antioxidant potential is dose dependent.

**Table 4:** The peroxide value and TBARS in obtained powders after 8 h of UV irradiation.

	Peroxide value (meq O_2_/kg of oil)	TBARS (µM MDA/g of powder)
Control powder non irradiated	< 0.8	0.80 ± 0.03 a
Control powder	69.10 ± 1.04 d	2.48 ± 0.11 d
Black cumin 0.5%	70.05 ± 0.23 d	2.15 ± 0.18 cd
Black sesame 0.5%	59.18 ± 0.57 c	2.13 ± 0.05 cd
Black cumin 1.0%	55.21 ± 0.06 b	2.07 ± 0.11 c
Black sesame 1.0%	47.74 ± 0.36 a	1.64 ± 0.07 b

a, b, … – Means in the same column for all variants followed by different letters are significantly different (*p* ≤ 0.05); Values are mean ± SD (*n* = 3).

Despite antiradical properties of melanin, it is also important that these compounds exhibit specifically anti-UV activity. It is commonly known that melanin found in the skin of animals and humans helps to protect skin cells (i.e. keratinocytes) from UV-induced DNA damage by shielding UV rays^53,54^. It has also been confirmed that melanin from fungi has a photoprotective effect^5^. Lower peroxide value observed in our study in most melanin-containing samples after UV irradiation may lead to the conclusion that plant melanin act against UV radiation similarly to those in human skin and can be an effective sunscreen ingredient for labile plant oils.

Previous research showed that peroxide value of freshly prepared powders was in the range 6.12 to 8.77 meq peroxide/kg oil and reached 110–140 meq peroxide after 4-week storage at elevated temperature^20^. It was stated that MD : WPC mixture was the most active material against lipid oxidation. Next study conducted by Hadad and Goli^21^ showed that microencapsulation using flax mucilage nanofiber was very effective in reducing the production of primary and secondary oxidative products compared to pure oil. Avramenko et al.^55^ reported that microencapsulation of the flaxseed oil by adding lentil protein and MD as an encapsulating agents improved the oxidative stability of the microencapsulated oil by 30 days (final peroxide value was ca. 50% lower in powder than in oil). Similarly, encapsulation with whey WPC and MD and sodium alginate process produced a gradual increase on the concentration of hydroperoxides; however, these values did not exceed the maximum allowed for cold pressed oils during storage^22^. Powders made with the use of crosslinked sodium caseinate with MD exhibited higher surface oil content after spray drying, and higher peroxide value^23^.

The content of thiobarbituric acid-reactive substances (TBARS) is presented in Table 4. The unirradiated control powder was characterized by a low TBARS content (0.80 µM/g). After UV irradiation, the content of secondary oxidation products increased in all powders, but in the samples with the added melanin preparation, these values ​​were significantly lower. Similarly to the peroxide value, the lowest TBARS content (1.64 µM/g) was recorded in the powder with 1% black sesame added. The obtained level of secondary oxidation products in irradiated flaxseed oil powders was like that found in various fried fast foods^56^.

### Induction period

The oxidative stability was analyzed using the Rancimat apparatus. This method may be conducted at various temperatures and measures the increase in conductivity of deionized water related to the solubilization of polar volatile compounds formed during oil oxidation. The results of the induction period (IP) of prepared powders are presented in Table 5. Pure flaxseed oil and control powder (without added melanin) had similar IP with values of 2.45 and 2.18 h, respectively. All powders with melanin were characterized by significantly higher IP values. The highest value (3.29 h) was obtained for 1% addition of black sesame melanin. In the case of powders with black cumin melanin, both variants showed statistically the same results.

**Table 5:** The induction period of obtained powders and flaxseed oil used in the study.

	Induction period (h)
Control oil	2.45 ± 0.14 a
Control powder	2.18 ± 0.08 a
Black cumin 0.5%	3.01 ± 0.11 bc
Black sesame 0.5%	3.10 ± 0.07 cd
Black cumin 1.0%	2.82 ± 0.04 b
Black sesame 1.0%	3.29 ± 0.05 d

a, b, … – Means in the same column for all variants followed by different letters are significantly different (*p* ≤ 0.05); Values are mean ± SD (*n* = 3).

In practice, only the surface oils are readily oxidized, since carbohydrates used as wall components usually provide a good oxygen barrier in spray-dried powders^57^. It was confirmed for example in the study by Shiga et al.^23^ with crosslinked sodium caseinate and MD. In the current study it was from 12.75 to 17.27% of surface oils which can be quickly oxidized to polar volatile compounds like aldehydes and ketones, like hexanal, heptan-2-one and octan-2-one^49,58^. In our previous study conducted with various mixtures of OSA starch with MD or nutriose or inulin the highest oxidative stability showed powders made with OSA starch and MD, but overall values of IP were relatively low (below 1 h)^19^. Generally, it is indicated that flaxseed oil has low oxidative stability due to its high content of unsaturated fatty acids. In this regard IP values determined for flaxseed oil powder higher than 50 h for oxidation in 110 °C^59^ seem unbelievable.

### Antioxidant capacity and activity of melanin in DPPH and ABTS assays

The rule of DPPH assay is that antioxidants in the sample react with DPPH to transform it to 1,1-dipheyl-2-(2,4,6-trinitropheyl) hydrazine due to the hydrogen donating ability. The scavenging potential of the antioxidants is determined based on their ability for discoloration measured at 515 nm^60^. Usually, antioxidant properties are presented as capacity (Trolox equivalent per sample mass), as free radical-scavenging activity (%) or as IC_50_ (extract concentration corresponding to 50% of antioxidant activity). In the current study results are presented as antioxidant capacity (Table 6). Capacity values were 19.70 and 4.38 µmol TE/g of sample for black cumin and black sesame extracts, respectively. Unfortunately, this level of antioxidant capacity was extremely low in relation to values of vitamin C (813 µmol TE/g).

**Table 6:** The antioxidant capacity of obtained melanin extracts in comparison to ascorbic acid.

	DPPH (µmol TE/1 g)	ABTS (µmol TE/1 g)
Black cumin melanin	19.7 ± 1.4 a	39.8 ± 3.5 a
Black sesame melanin	4.38 ± 0.78 a	93.7 ± 0.0 b
Ascorbic acid	813 ± 60 b	186.9 ± 13.9 c

a, b, … – Means in the same column for all variants followed by different letters are significantly different (*p* ≤ 0.05); Values are mean ± SD (*n* = 3).

Previous research showed that the DPPH scavenging activities of L-25 melanin (from sorghum black powder fungus) exhibited a dose–response relationship. At a concentration of 0.4 mg/mL, the hydroxyl radical scavenging rate of L-25 melanin reached 76.86%^61^. Similarly, the DPPH radical scavenging activity of the melanin extracted from *Gluconobacter oxydans* FBFS 97 increased with increasing concentrations and exhibited a dose–response relationship. At a concentration increase from 1.56 to 6.25 µg/mL, the DPPH-scavenging rate of extracted melanin increased from 1.2% to 7.58%^60^.

In the ABTS assay stable ABTS radical cation is terminated by antioxidants present in the tested sample. This mechanism utilizes primarily single electron transfer^62^. Antioxidant capacity values were 39.8 and 93.7 µmol TE/g of sample for black cumin and black sesame extracts (Table 6). These values were significantly higher than obtained for DPPH assay what suggest that melanin preparations are a better donor of single electron than hydrogen atom. Black sesame melanin preparation was a greater electron donor than black cumin, but both these preparations exhibited significantly lower antioxidant capacity in comparison to ascorbic acid (186.9 µmol TE/g).

## Conclusions

Melanin can be effectively extracted from seeds or from by-products of black cumin and black sesame processing. Melanin preparations from these sources differ in color, with significantly more yellow shade of black cumin preparation. The UV-VIS spectra of melanin preparations varied in the range of 250–300 nm, indicating possible contamination especially in the black sesame extract. Addition of 0.5 and 1% of melanin to wall material did not change powders particle size and shape, but slightly affected color and flowability of powders (in relation to control sample) and significantly affected oxidation of flaxseed capsules. 1% addition of black sesame melanin protected flaxseed oil before oxidation in Rancimat assay and during UV irradiation monitored by peroxide value and TBARS measurement. Both types of melanin showed activity against the DPPH and ABTS radicals, but the level of this protection was much lower than in the case of vitamin C. It suggests that effect observed in UV-irradiation test was mainly caused by melanin light screen shielding properties rather than radical scavenging.

To summarize the research results, melanin preparations from black cumin and black sesame seeds can be used as natural antioxidants in the process of encapsulation of flaxseed oil. 1% melanin in the wall material during the encapsulation process appears to be an effective agent of protecting this unstable oil, but further research is needed to determine the most effective percentage. Additionally, storage studies of such powders and application studies in various food matrices enriched with flaxseed ALA are necessary. The protective influence of melanin should be monitored at various light wavelengths and compared to other known food-grade UV shielding agents, like carotenoids, polyphenols, etc.

## Data Availability

Data created during the current study is available from the corresponding author on reasonable request.
